# Vibratory Reaction Unit for the Rapid Analysis of Proteins and Glycochains

**Published:** 2007-09-17

**Authors:** Yukie Sasakura, Makoto Nogami, Noriko Kobayashi, Katsuhiro Kanda

**Affiliations:** 1 Bio-Medical Center, R&D Division, Nanotechnology Product Business Group, Hitachi High-Technologies Corporation, Hitachinaka, Ibaraki, 312-8504, Japan.; 2 Naka Application Center, Nanotechnology Product Business Group, Hitachi High-Technologies Corporation, Hitachinaka, Ibaraki, 312-0057, Japan.

**Keywords:** mass spectrometry, immobilized enzyme, protein, glycochain, micro scale reaction

## Abstract

A protein digestion system using immobilized enzymes for protein identification and glycochain analyses has been developed, and a vibration reaction unit for micro-scale sample convection on an enzyme-immobilized solid surface was constructed. BSA as a model substrate was digested by this unit, and was successfully identified by mass spectrometry (MS) analyses. Compared to the conventional liquid-phase digestion, the reaction unit increased the number of matched peptides from 9 to 26, protein score from 455 to 1247, and sequence coverage from 21% to 48%. Glycopeptidase F (NGF), an enzyme that cleaves N-glycans from glycoproteins, was also immobilized and used to remove the glycochains from human immunoglobulin G (IgG). Trypsin and NGF were immobilized on the same solid surface and used to remove glycochains from IgG in single-step. Glycochains were labeled with fluorescent reagent and analyzed by HPLC. Several peaks corresponding to the glycochains of IgG were detected. These results suggested that the single-step digestion system, by immobilized multiple enzymes (trypsin and NGF) would be effective for the rapid structural analysis of glycoproteins.

## Introduction

Analyses of the protein structure and post-translational modification (PTM) such as glycochain, are one of the most important issues in biomarker discovery and drug development. High-performance liquid chromatography (HPLC) and mass spectrometry (MS) have been widely used as powerful tools for the characterization and identification of proteins and glycochains. (Reinders, 2004) (Hirabayashi, 2002).

For these analyses, digestion of proteins into peptides or removal of glycochains from glycoproteins is required. Usually, these reactions need overnight incubation, interrupting the high throughput analysis that is essential for large scale proteomics or glycomics analyses. Even when such a long reaction is performed, the digestion efficiency is often insufficient, resulting in poor identification or characterization of target proteins. Especially in PTM analyses, complete digestion is strongly required, because if the peptides corresponding to the PTM site were not generated, it would be difficult to detect and identify it.

For the analyses of glycochain attached to the proteins, glycoprotein is first fragmented to peptide by protease, and then treated by glycosidase to remove its glycochains. Isolated glycochains are usually labeled by fluorescent dye, and analyzed by single or multi-dimensional HPLC, or MS. (Kakehi, 1993; Morelle, 2006). Treatment of proteases and glycosidases is generally performed sequentially, leading to a time consuming and complicated experiment.

In this study, we developed two high-throughput sample preparation methodologies using enzyme-immobilized slide glass and the reaction unit for the micro-scale sample convection; protein digestion system for the protein identification and glycochain removal system for the glycochain analyses. In the former system, three kinds of protease-immobilized chips (trypsin, chymotrypsin, and ArgC were immobilized, respectively) were prepared and used to digest BSA as a model substrate. In the latter system, trypsin and glycopeptidase F (NGF), an enzyme that cleaves N-glycans from glycoproteins, were immobilized on the same glass surface and used to remove the glycochains from human immunoglobulin G (IgG).

## Experimental

### Materials

The vibration reaction unit was manufactured by Fluidware Technologies (Tokyo, Japan). The ProteoChip^™^ was obtained from Proteogen (Seoul, Korea), and hydrophobic seal was purchased from Nakagawa Chemical (Tokyo, Japan). Trypsin, chymotrypsin, and ArgC were obtained from Sigma-Aldrich (St. Louis, MO), Calbiochem (San Diego, CA), and Roche (Basel, Switzerland), respectively. HybriWell^™^ sealing system was purchased from GRACE BIO-LABS (Bend, OR). BSA and IgG were obtained from Sigma-Aldrich, and NGF was purchased from Roche. Isomaltoheptaose and ABOE labeling kit was obtained from SEIKAGAKU CORPORATION (Tokyo, Japan). Other chemicals were obtained from Wako Pure Chemicals (Osaka, Japan) and Sigma-Aldrich.

### Preparation of the protease-immobilized chip

For the preparation of the enzyme-immobilized chip, ProteoChip^™^ was used as a solid substrate. Three kinds of protease solutions (trypsin, chymotrypsin, and ArgC) were prepared at 1 mg/ml with PBS (pH 7.4). HybriWell^™^ sealing system containing six incubating chambers was adhered to the ProteoChip^™^ surface in advance. The protease solutions were each introduced into the different chambers and incubated overnight at 4°C for the immobilization on the surface. The chip was immersed in PBS (pH 7.8) with gentle shaking for removal of unbound proteases, rinsed with 10 mM Tris-HCl (pH 8.0) and dried using a spin dryer prior to the digestion.

### Reduction and alkylation of the proteins

1 mg of BSA or IgG was diluted to 1 ml of denaturing buffer (200 mM Tris-HCl, pH 8.5, containing 6M Guanidine-HCl and 2.5 mM EDTA). After addition of 60 μg of DTT, the surfaces of the solutions were blown by N_2_ gas, and incubated for 3 h at 37 °C. After incubation for 5 min on ice, 1 mg of iodoacetamide was added, the surfaces of the solutions were blown by N_2_ gas, and then incubated for 1 h at RT under dark condition. Guanidine-HCl was removed by dialysis against 10 mM Tris-HCl (pH 8.0).

### HPLC analyses of the digested peptides

HPLC analyses were performed using Hitachi L-2000 system (Hitachi High-Technologies, Tokyo, Japan). Digested samples were applied to a CAPCELLPAK C_18_ MG column (2 mm I.D. × 75 mm, Shiseido, Tokyo, Japan). Two buffers were prepared as the mobile phase: 5% acetonitrile/95% water containing 0.1% formic acid (buffer A) and 95% acetonitrile/5% water containing 0.1% formic acid (buffer B). Elution was performed using a linear gradient of 100:0 to 60:40 A/B delivered at 0.2 mL/min over 50 min. The absorption at 214 nm was detected.

### Preparation of the trypsin/NGF immobilized chip

Trypsin solution was prepared at 1 mg/ml with PBS (pH 7.4), and NGF solution was prepared at 30 μg/ml with 100 mM phosphate buffer containing 25 mM EDTA. Hydrophobic sheet (25 mm × 65 mm) containing three holes (17 mm × 15.5 mm) was adhered in advance to the ProteoChip^™^. 60 μl of NGF solution was added to the center hole and 60 μl of trypsin solution was added to the two side holes. The chip was incubated overnight at 4 °C for the immobilization on the surface.

### Removal of glycochains from human IgG (conventional method)

50 μg of trypsin was added to the 1 mg of reduced and alkylated human IgG, and incubated overnight at 37 °C. After denaturation of the enzyme by boiling, 80 ng of NGF was added and incubated overnight at 37 °C.

### Purification and *p*-aminobenzoic acid octyl ester (ABOE)-labeling of the isolated glycochains

Digested IgG solution was lyophilized, dissolved with 10 μl of sterile water, and 2 μg of isomaltoheptaose was added as an internal standard. This was labeled with ABOE using ABOE Labeling Kit, and concentrated by lyophilization.

### HPLC analyses of the ABOE-labeled glycochains

ABOE-labeled glycochains were applied to a Honenpak C_18_ column (4.6 mm I.D. × 75 mm, SEIKAGAKU CORPORATION). Two buffers were prepared as the mobile phase: 75% 0.1M ammonium acetate (pH 4)/25% acetonitrile (buffer C) and 60% 0.1M ammonium acetate (pH 4)/40% acetonitrile (buffer D). After flow of buffer C for 10 min, elution was performed using a linear gradient of 100:0 to 50:50 C/D delivered at 1 mL/min over 40 min. Fluorescence of ABOE was detected (excitation at 330–350 nm, emission at 410 nm).

## Results and Discussion

### The vibration reaction unit

**Figure 1:**
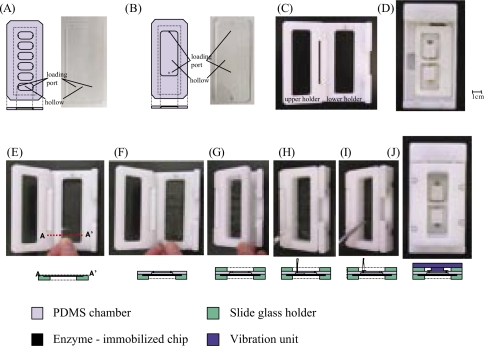
The vibration reaction unit. (A) The PDMS chamber Type 1 containing six hollows (50 μl volume) and (B) Type 2 containing one hollow (0.2 ml), that may be filled with samples and reagents on the slide glass surface. Sample loading ports were attached to each hollow. Their designs (left) and pictures (right) are shown. (C) The slide glass holder is composed of upper and lower holders that are used to support the slide-affixed PDMS chamber. (D) The vibration unit can cause convection within the hollows. The operation of the vibration reaction unit is shown in (E)~(J). (E) The enzyme-immobilized chip is placed on the lower-holder. (F) The enzyme-immobilized chip is covered by the PDMS chamber. (G) The holder is closed to clamp the PDMS chamber and the enzyme-immobilized chip. (H) The sample is loaded through the sample loading port. (I) The sample loading ports are sealed with the small pieces of PDMS seat. (J) The vibration unit is attached for the sample agitation.

The vibration reaction unit (Kuno, 2003; Sasakura, 2006) includes PDMS chambers (Fig. 1(A), (B)), a slide glass holder (Fig. 1(C)) and a vibration unit (Fig. 1(D)). PDMS is considered an excellent material for use in microfluidic systems targeted towards analysis of biological samples (Sia, 2003) (McDonald, 2000), as it is inexpensive, impermeable to water, nontoxic to biomolecules, and able to attach onto glass surfaces. Here, two types of PDMS chambers were prepared; the PDMS chamber Type1 consists of six small hollows (50 μl volume, Fig. 1(A)) and Type 2 consists of one hollow (0.2 ml volume, Fig. 1(B)), with sample loading ports. After the chamber was attached to the slide glass surface to generate the reaction baths in the hollows (Fig. 1(F)), they were set on the slide glass holder (Fig. 1(G)), samples or reagents were injected via the sample loading ports using a pipette (Fig. 1(H)), the ports were sealed (Fig. 1(I)), and the vibration unit was attached to cause convection within the reaction baths (Fig. 1(J)).

### Protein digestion by protease-immobilized chip

**Figure 2:**
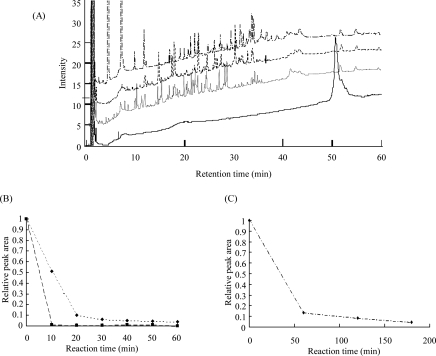
BSA was treated with three kinds of protease-immobilized chips using the vibration reaction unit. Digested samples were analyzed by HPLC and generated peptides were detected. (**A**) The HPLC chromatograms of undigested control (solid line,) trypsin, chymotrypsin, and ArgC-digested samples (dotted, dashed and dashed-dotted line, respectively) are shown. (**B, C**) Their time-dependent digestions were analyzed. Their digestion rates were calculated from the relative peak area of the undigested BSA, which was shown at the retention time of 50 min. Digestion efficiency of trypsin, chymotrypsin ((**B**) dotted and dashed line, respectively), and ArgC (**C**) reached over 90% within 20 min, 10 min, and 2 hr, respectively.

We prepared three kinds of protease-immobilized chips (trypsin, chymotrypsin, and ArgC were immobilized, respectively). BSA was digested using these chips with the reaction unit, and analyzed by HPLC. Their HPLC chromatograms are shown in Figure 2(A). Their digestion rates were calculated from the relative peak area of the undigested BSA, which was shown at the retention time of 50 min. The time-dependent analyses of the digestion are shown in Fig. 2(B) (trypsin and chymotrypsin) and (C) (ArgC). Digestion efficiencies of the immobilized trypsin, chymotrypsin, and ArgC reached over 90% within 20 min, 10 min, and 2 hr, respectively.

**Figure 3:**
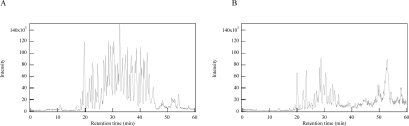
Tryptic digested BSA was analyzed by MS. The total ion chromatograms of the sample digested by the vibration reaction unit (**A**) and conventional liquid-phase reaction (**B**) are shown.

The protein identification using MS is largely dependent on the protein digestion; insufficient digestion often results in poor identification, because the proteins will not match the peptides listed in the database. Therefore, proteins should be completely fragmented before MS analyses. The total ion chromatogram of the tryptic-digested proteins is shown in Figure 3(A). The peak of undigested protein disappeared, which was shown in the conventional liquid-phase digestion at 50 min of the retention time (Fig. 3(B)). As a result, digestion by the vibration improved the number of matched peptides from 9 to 26, protein score from 455 to 1247, and sequence coverage from 21% to 48% (Table 1). When completely digested BSA (after overnight digestion) was analyzed at the same MS conditions, the sequence coverage is usually around 50%. The 100% of coverage could not been obtained, because of the differences of ionozation or detection efficiency of each peptides. These results suggested that 1hr digestion using our new method could give the same results obtained by the conventional overnight digestion. Therefore, protease-immobilized chip and micro-scale sample agitation are effective for high-throughput protein structural analysis.

There have been a lot of reports about protein digestion systems using immobilized trypsin. (Massolini, 2005) However, the digestion system using other proteases, such as chymotrypsin or ArgC has not been focused, even though their importance for accurate protein structural analysis has been suggested (Gatlin, 2000). Particularly, the PTM analyses often require a different type of fragmentation depending on the modification sites. Therefore, several kinds of proteases should be considered, and this work should be effective for these purposes.

### Glycochain analysis using Trypsin/NGF-immobilized chip

For the analyses of glycochain attached to the proteins, glycoproteins are first fragmented to peptides by protease and treated by glycosidase to release the glycochain. Isolated glycochains are usually labeled with fluorescent dye, and analyzed by HPLC. The retention times of glycochains are compared to that of known standard glycochains, and their structural character can be estimated, (Kakehi, 1993).

Treatment of proteases and glycosidases is usually performed sequentially. First, the samples are treated with proteases and then, proteases are denatured by boiling. Next, they are treated by glycosidases and released glycochains are collected. This sequential-step treatment is troublesome and time consuming.

Here, we prepared an enzyme immobilized chip containing two enzymes (trypsin and NGF) on the same surface, and used it to perform fragmentation of the protein followed by release of glycochains. Generation of peptides was confirmed by HPLC. Collected glycochains were labeled with fluorescent dye (ABOE), and analyzed by HPLC.

**Figure 4:**
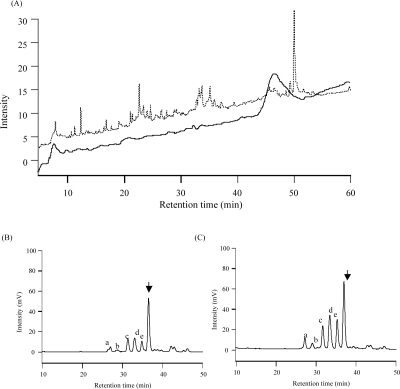
Human IgG was treated by trypsin/NGF-immobilized chip. (**A**) Generation of peptides was confirmed by HPLC. The chromatograms of undigested control (solid line), and digested samples (dotted line) are shown. (**B**)(**C**) Fluorescence-labeled glycochains removed from IgG were analyzed by HPLC. The HPLC chromatograms of the samples digested by the vibration reaction unit (**B**) and conventional liquid-phase reaction (**C**) are shown.

Figure 4(A) shows the HPLC chromatogram of IgG peptides. The disappearance of the intact protein’s peak and generation of the peptides’ peaks were confirmed. When it was analyzed by MS, several human IgGs were identified with enough sequence coverage, suggesting that this method can be used for the structural analysis of glycoprotein. Figure 4(B) shows the HPLC chromatogram of ABOE-labeled glycochains from IgG. A peak indicated by the arrow corresponds to the isomaltoheptaose, which was added as an internal standard. All other peaks are originated from the IgG. Their relative peak areas were normalized so that the internal standard intensity was 1 (Table 2). The retention time of each glycochain was consistent with that obtained in the conventional liquid-phase digestion (Fig. 4(C)), suggesting the same glycochains were collected by our new method. In addition, single-step digestion using trypsin and NGF immobilized chip reduced the experimental time from 2 days to 1 day, suggesting the efficiency of this method for high-throughput glycochain analysis.

For clinical or diagnostic purposes, the differential analysis of glycochains between patients and healthy controls generates great interest. In this case, the relative quantity of each glycochain is important, because it could reflect the state of a disease. For this reason, the reproducibility of the glycochain quantity is important. The quantities of each glycochain (a ~ e in Fig. 4 (B)(C)) obtained in this experiment were 50%, 59%, 71%, 58%, 55% of that obtained in the conventional liquid-phase reaction, suggesting that there would be differences between digestion pattern of our new method using immobilized enzymes and the conventional liquid phase method. This might be caused by differences in accessibility of NGF to glycosilation sites, which is dependent on the structure of the glycoprotein. However, if we can obtain the same digestion rate reproducibly and distinguish the differences of the glycochain contents, we can adapt our method to the differential analysis of glycoproteins for diagnostic or clinical purposes. Confirming the reproducibility of our new methods and adapting them to the diagnostic test or other clinical purposes is our next task.

There are a few reports about immobilized NGF for glycochain analysis. Especially, the one-step reaction with trypsin on a single solid surface is a novel technique, and has become an effective sample preparation method for glycochain analysis. The fact that immobilized two enzymes do not interact each other, enables them to be active in the single reaction. Now, the 2D-mapping method using HPLC is most widely used for glycochain identification. (Kakehi, 1993) However, analysis using MS has recently received a lot of attention (Morelle, 2006), because it can determine the structures of glycochains directly. Our novel system could also be adopted for the MS-based glycoprotein analysis.

Compared to the conventional liquid-phase digestion, immobilized enzymes have several advantages; no contamination of samples with the enzymes, increased enzyme/substrate ratio, and reusability. On the other hand, the main disadvantage in this study is that a large amount of enzyme is required for the preparation of an enzyme-immobilized chip, increasing the cost for the analysis. Therefore, adjusting the amount of enzyme used to prepare the enzyme-immobilized chip will be our next challenge.

## Conclusion

In this study, protein digestion methodologies using immobilized enzymes and the microscale vibration reaction unit have been developed. BSA was digested by a trypsin-immobilized chip within 20 min, chymotrypsin-immobilized chip within 10 min, and ArgC-immobilized chip within 2 h. In addition, the reaction unit improved the number of matched peptides, the score, and the sequence coverage for the protein identification. The reaction unit could also perform protein digestion and glycochain removal from the glycoproteins in a single-step. These results suggest that the digestion system using immobilized enzymes and the vibration reaction unit would be effective for the rapid analysis of proteins and glycochains.

## Figures and Tables

**Table 1:** Results of MS analyses of tryptic digested BSA.

	**The reaction unit**	**Liquid-phase**
Peptides matched	26	9
Protein score	1247	455
Sequence coverage	48%	21%

**Table 2:** Relative quantity of the isolated glycochains.

		**Peaks**					
		**IgG^*a*^**					**Internal standard**
		**a**	**b**	**c**	**d**	**e**	
Relative	(B) The reaction unit	0.08	0.07	0.24	0.31	0.21	1.00
peak area*^b^*	(C) Liquid-phase	0.17	0.12	0.34	0.53	0.38	1.00
	(B)/(C) × 100	50%	59%	71%	58%	55%	100%

*^a^*The corresponding peaks are shown in Figure 4 (B)(C).

*^b^*They were calculated so that the internal standard intensity was 1.

## Abbreviations:

BSA: bovine serum albumin
MS: mass spectrometry
NGF: glycopeptidase F
IgG: immunoglobulin G
PTM: post-translational modification
HPLC: high-performance liquid chromatography
PBS: phosphate-buffered saline
EDTA: ethylenediaminetetraacetic acid
DTT: dithiothreitol
RT: retention time
ABOE: *p*-aminobenzoic acid octyl ester
PDMS: polydimethylsiloxane
ArgC: endoprotease Arginine C
